# The rapid growth of bibliometric studies: a call for international guidelines

**DOI:** 10.1097/JS9.0000000000001049

**Published:** 2024-01-11

**Authors:** Kunming Cheng, Zhiyong Li, Zaijie Sun, Qiang Guo, Wanqing Li, Yanqiu Lu, Shaoyan Qi, Zefeng Shen, Ruijie Xie, Yulin Wang, Zhenjiang Wu, Yinhui Wu, Chunchun Wu, Yuqiao Li, Yuchen Xie, Haiyang Wu, Cheng Li

**Affiliations:** a Department of Intensive Care Unit, The Second Affiliated Hospital of Zhengzhou University; b Department of Thoracic Surgery, Henan Provincial Chest Hospital, Zhengzhou University, Zhengzhou; c Department of Orthopaedic Surgery; d Department of Operating Room, Xiangyang Central Hospital, Affiliated Hospital of Hubei University of Arts and Science, Xiangyang; e Department of Urology, Peking University Shenzhen Hospital, Shenzhen; f Department of Microsurgery, The Affiliated Nanhua Hospital, Hengyang Medical School, University of South China, Hengyang; g Xiangya Medical College, Central South University, Changsha, Hunan; h Department of General Practice, Zhoukou Central Hospital; i Department of Emergency, Taikang People’s Hospital, Zhoukou, Henan; j Department of Orthopedics, Baodi Clinical College of Tianjin Medical University; k Department of Clinical College of Neurology, Neurosurgery and Neurorehabilitation, Tianjin Medical University, Tianjin; l Department of Orthopaedic Surgery, Peking University People’s Hospital; m Department of Orthopaedic Surgery, Beijing Jishuitan Hospital, Capital Medical University, Beijing, People’s Republic of China; n Duke Molecular Physiology Institute, Duke University School of Medicine, Durham, North Carolina, USA; o Division of Clinical Epidemiology and Aging Research, German Cancer Research Center (DKFZ), Heidelberg; p Center for Musculoskeletal Surgery (CMSC), Charité-Universitätsmedizin Berlin, corporate member of Freie Universität Berlin, Humboldt University of Berlin, and Berlin Institute of Health, Berlin, Germany

*To the Editor*,

Over the past decade, the fields of basic science and clinical medicine have undergone a significant transformation due to the rapid development of information technology and the widespread adoption of the Internet. This transformation has led to an unprecedented ‘information explosion’ revolution, as evidenced by pouring out a huge number of scientific publications and electronic databases. In academic activities, scholars are confronted with the pressing challenge of effectively managing and updating their knowledge base to keep pace with the rapid emergence of new trends and research hotspots. Traditional literature reviews, solely relying on manual screening and sorting of literature, have proven inadequate in handling the overwhelming influx of scientific publications. Therefore, it is particularly crucial to leverage bibliometrics, first defined by Pritchard in 1969, which take advantage of bibliometric theory utilizing mathematical and statistical methods, to swiftly and accurately process vast amounts of academic literature.

Bibliometric methods offer researchers the ability to gain deeper insights and perform comprehensive analyses of the extensive body of scientific literature. By utilizing these methods, researchers can rapidly screen, classify, and organize extensive amounts of literature information, enabling the discovery of new research fields and the identification of emerging hotspots. Through the visualization of literature data, researchers are provided with a clear view to observe and analyze scientific research trends, hot topics, and knowledge networks. Additionally, these methods facilitate the identification of significant information such as research dynamics within specific fields, collaborative networks among authors, and the impact of research outcomes. Furthermore, bibliometric analysis allows researchers to understand the position and influence of their own research within the broader context of the field. By examining citation relationships between documents, author collaboration networks, and impact indicators of research outcomes, researchers can gain valuable insights into rapidly developing research fields and areas of notable interest. Such insights serve as essential guidance for researchers in selecting research directions, formulating effective research strategies, and securing research funding.

In recent years, a surge of bibliometric articles has emerged in various journals, including the *International Journal of Surgery* ^1–4^. There has been a rapid increase in the number of articles focusing on bibliometric analysis. Bibliometric methods have gradually attracted the attention of more and more scholars. To explore the global publication trend related to bibliometric studies, our investigation involved the retrieval of pertinent studies on bibliometrics from the Web of Science Core Collection database as of 16 December 2023. The search encompassed titles (TI), abstracts (AB), and author keywords (AK) employing the terms ‘bibliometric*’ or ‘scientometric*’. Our selection criteria were limited to English-language publications between 2000 and 2023, encompassing original articles and reviews. Following manual deduplication, we obtained a final dataset comprising 16 388 unique records, comprising 11 358 articles and 5030 reviews. As for whether bibliometric analysis is a review or an original article, different journals or editors have different views. Overall, it can be seen that 69% of bibliometric studies are classified as original articles. The reason for this result might be due to the process of bibliometric analysis involving data extraction and analysis, which is quite different from system reviews.

The annual number of bibliometric-related publications from 2000 to 2023 is shown in Figure 1A. The publication outputs have increased from 51 in 2000 to 3434 in 2022, and up to 16 December 2023, the total number of publications has reached 2931. The model fitting curve revealed an exponentially increasing trend over the past 24 years (R2=0.9375). The rapid growth of bibliometric articles may be attributed to several factors. Firstly, there is an increasing emphasis in the academic sphere on evaluating research quality and impact. Bibliometric analysis provides an objective approach to measure indicators such as the impact and citation status of research outcomes. Secondly, advancements in computer and database technologies have made it easier to access and analyze large-scale literature data. The expansion of literature databases and the promotion of open access have further encouraged researchers to use extensive literature datasets. Moreover, some countries and institutions consider bibliometric analysis as a reference for policymaking and resource allocation. This policy orientation likely encourages more researchers to engage in bibliometric analysis to better comprehend trends in academic fields and aid in policy formulation. Therefore, it is foreseeable that the number of articles related to bibliometric analysis will continue to increase in the future.

**Figure 1:**
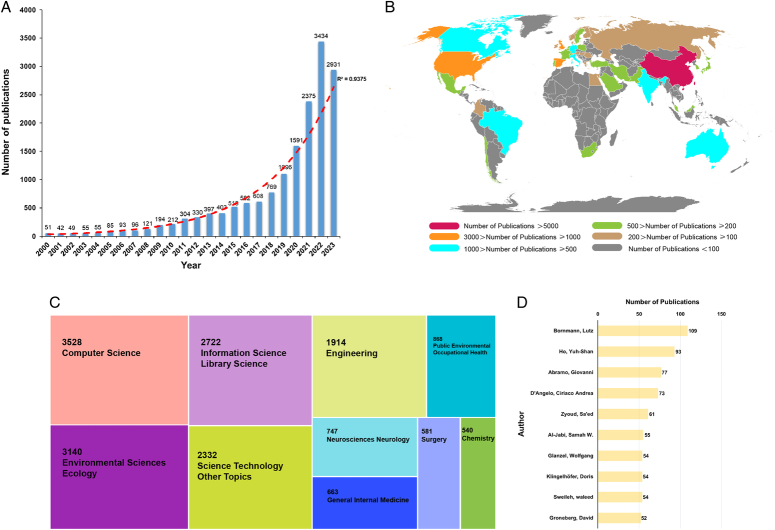
(A) The annual number of bibliometric-related publications from 2000 to 2023; (B) geographical distribution of global publications; (C) top 10 research areas categorized by their respective publication counts; (D) top 10 authors categorized by publication outputs.

As for the major contributors in the bibliometric field, all the publications originated from 152 countries/regions. Of note, Figure 1B highlights the geographical distribution of global publications. Among them, there is no doubt that China has occupied the dominant position, accounting for more than one-third of all publications (*n*=5873), followed by the United States (*n*=2252, 13.7%) and Spain (1305, 8.0%). When it comes to authors (Fig. 1D), Bornmann Lutz from Max Planck Society emerged as the most prolific author, followed by Ho Yuh-Shan from Asia University Taiwan and Abramo Giovanni from the University of Rome Tor Vergata. In addition, we also summarized the top 10 research areas categorized by their respective publication counts in Figure 1C. Computer Science, Environmental Sciences Ecology, as well as Information Science Library Science were the top three research areas that received the most attention. In terms of the domain of biomedicine, bibliometrics has more applications in the fields of Neurosciences Neurology, General Internal Medicine, and Surgery. However, there is still a significant gap compared with other disciplines, which indicates its huge potential in biomedicine.

Of note, one significant issue is that despite the exponential growth of bibliometrics articles currently, there is still no universally accepted international guideline to direct the writing of bibliometric analyses. As a result, there exists considerable disparity in the retrieval methods, search term settings, literature screening, and analytical processes among many studies^5,6^. Even when secondary analyses are conducted following the authors’ described methods, disparate outcomes may arise. Simultaneously, to our knowledge, the *International Journal of Surgery* handles submissions related to bibliometric analysis as reviews and requires authors to submit the Preferred Reporting Items for Systematic Reviews and Meta-Analyses (PRISMA) 2020 Checklist^7^. However, the PRISMA 2020 Checklist is not suitable for bibliometric analysis, as several elements within the checklist do not align with aspects pertinent to bibliometric analysis. Moreover, critical aspects of bibliometric analysis methodology, notably database sources, search term settings, data screening processes, and analytical procedures, cannot be adequately reflected or documented using this checklist. Our research team is currently engaged in revising the PRISMA 2020 Checklist to better suit the application of bibliometric analysis. Subsequent outcomes from this endeavor are pending publication.

In summary, bibliometric analysis is a swiftly evolving field, but this rapid advancement necessitates guidance via standardized guidelines. We advocate for increased participation of scholars in formulating rational guidelines specific to bibliometric analysis, thereby promoting standardization within the field.

## Ethical approval

This study does not include any individual-level data and thus does not require any ethical approval.

## Sources of funding

This study is supported by China Postdoctoral Science Foundation (2022M720385) and Beijing JST Research Funding (YGQ-202313).

## Author contribution

K.C.: conceptualization, methodology, data curation, formal analysis, resources, investigation, and writing – original draft; Z.L., Q.G., Z.S., W.L., Y.L., S.Q., Z.S., R.X., Y.W., Z.W., Y.W., C.W., Y.L., Y.X., and H.W.: conceptualization, methodology, data curation, formal analysis, resources, and investigation; C.L.: methodology, data curation, formal analysis, resources, investigation, and writing – review and editing.

## Conflicts of interest disclosure

The authors declare no conflicts of interest.

## Research registration unique identifying number (UIN)

1. Name of the registry: not applicable.
2. Unique identifying number or registration ID: not applicable.
3. Hyperlink to your specific registration (must be publicly accessible and will be checked): not applicable.

## Guarantor

Haiyang Wu and Cheng Li.

## Data availability statement

The data underlying this article will be shared by the corresponding author on reasonable request.

## References

[1] Zhu W Ding X Zheng J. A systematic review and bibliometric study of Bertolotti’s syndrome: clinical characteristics and global trends. Int J Surg 2023;109:3159–3168. 37318877 10.1097/JS9.0000000000000541 PMC10583961

[2] Zhang X Yi K Xu J-G. Application of three-dimensional printing in cardiovascular diseases: a bibliometric analysis. Int J Surg 2023. doi:10.1097/JS9.0000000000000868 PMC10871659 37924501

[3] De Felice F Cattaneo CG Poto GE. Mapping the landscape of immunonutrition and cancer research: a comprehensive bibliometric analysis on behalf of NutriOnc Research Group. Int J Surg 2023. [Epub ahead of print]. doi:10.1097/JS9.0000000000000783 PMC10793798 37737933

[4] Pan Y Deng X Chen X. Bibliometric analysis and visualization of research trends in total mesorectal excision in the past twenty years. Int J Surg 2023;109:4199–4210. 37678311 10.1097/JS9.0000000000000681 PMC10720803

[5] Cheng K He Y Gu S. A commentary on ‘Evolutionary patterns and research frontiers in neoadjuvant immunotherapy: a bibliometric analysis’. Int J Surg 2023;109:2829–2830. 37352515 10.1097/JS9.0000000000000529 PMC10498845

[6] He Y Tang H Wu H. Comments on ‘Insight into the history and trends of surgical simulation training in education: a bibliometric analysis’. Int J Surg 2023;109:3228–3229. 37335983 10.1097/JS9.0000000000000547 PMC10583962

[7] Page MJ McKenzie JE Bossuyt PM. The PRISMA 2020 statement: an updated guideline for reporting systematic reviews. Int J Surg 2021;88:105906. 33789826 10.1016/j.ijsu.2021.105906

